# Causes of Diphtheria

**Published:** 1879-05

**Authors:** Henry Mills

**Affiliations:** Microscopist


*ART. II.—Causes of Diphtheria.* By MILLS.

Among the many theories as to the cause of diphtheria, I notice a statement of Dr. Quesner in a recent number of the *Boston Chemical News*, that he has almost come to the conclusion that the disease is to be traced to a fungus found in spots on fruits, especially apples. The fact that the disease was very prevalent last summer and fall in many of the apple growing districts of our own and other States, would seem to give weight to this decision. He says during the summer and fall, children are in the daily habit of picking fruit from the ground and eating immediately without rubbing or cleansing, thus taking into the system whatever may have grown or become attached to the surface of the fruit.

The fungoid origin of whooping cough was asserted some years since by M. Svetzerick, and seems now to be confirmed by M. Yschamer, who says he has found certain lower organisms in the spittle of whooping cough patients—organisms not met with in any other disease accompanied with cough and expectoration. He claims further, that the organisms are identical with those which by their agglomeration, form the black points or spots on the skin of many fruits, especially apples. M. Yschamer by inoculating rabbits with this fungus produced similar result to whooping cough.

The writer of this article as a microscopist has examined fruit this winter and has found the black spots on apples to consist of a fungus--one of the oidia. The spots vary in size from a pins head to a good sized pea. A small portion of the dark, downy tuft growing in the center of the spot when removed by the point of a pen-knife to a suitable slide and placed under the microscope, will show the fungus. Mycelium and threads and spores extending from these sometimes cover a large part of the apple as may be seen with even a low power of the instrument. Slides can easily be prepared so as to show the fungus either as opaque or transparent. The spores are inconceivably small, and thousands of them with the mycelium might lodge in the fauces without being felt, unless they should begin to germinate.

The microscope reveals the fact that the decayed part of apples also is infested through and through with a similar fungus. Mr. Thomas Taylor, a microscopist in Washington, D. C., has discovered an Entozoa one of the anguellulæ in a diseased pear, also in diseased peaches, and even in a diseased trophy tomato. These remarks are not intended to discourage the plentiful use of good ripe and sound fruit, but from the facts stated, it must appear to be the duty of all who value their own health and the health of those depending on them to see that the fruit eaten is perfectly sound and clean out side and in.

